# Randomized controlled trial comparing the effectiveness of the ultrasound-guided galvanic electrolysis technique (USGET) versus conventional electro-physiotherapeutic treatment on patellar tendinopathy

**DOI:** 10.1186/s40634-016-0070-4

**Published:** 2016-11-16

**Authors:** F. Abat, J. L. Sánchez-Sánchez, A. M. Martín-Nogueras, J. I. Calvo-Arenillas, J. Yajeya, R. Méndez-Sánchez, J. C. Monllau, P. E. Gelber

**Affiliations:** 1 Department of Orthopaedic Sports Medicine, ReSport Clinic, Rambla Fabra i Puig 47, 08030 Barcelona, Spain; 2 Department of Physiotherapy, University of Salamanca, Campus Miguel de Unamuno, 37007 Salamanca, Spain; 3 Department of Physiology, University of Salamanca, Campus Miguel de Unamuno, 37007 Salamanca, Spain; 4 Catalan Institut of Traumatology and Sports Medicine (ICATME), Hospital Universitari Dexeus, Universitat Autónoma de Barcelona, Sabino Arana 5-19, 08028 Barcelona, Spain; 5 Department of Orthopedic Surgery and Traumatology, Parc de Salut Mar, Universitat Autónoma de Barcelona, Passeig Marítim, 25-29, 08003 Barcelona, Spain; 6 Department of Traumatology and Orthopaedic Surgery, Hospital de la Santa Creu i Sant Pau, Universitat Autónoma de Barcelona, Sant Quintí 89, 08041 Barcelona, Spain

**Keywords:** USGET, Galvanic, Electrolysis, Treatment, Patellar, Tendinopathy, Electrophysiotherapy

## Abstract

**Background:**

Patellar tendinopathy has a high prevalence rate among athletes. Different therapeutic options can be found in the current literature, but none of them has been clearly established as the gold standard. The purpose of this study is to compare, in a randomized controlled trial, the clinical efficacy of eccentric exercise combined with either an ultrasound-guided galvanic electrolysis technique (USGET) or conventional electrophysiotherapy to treat patellar tendinopathy.

**Methods:**

Sixty patients diagnosed with patellar tendinopathy were randomized into two groups. Group 1 (*n* = 30) received electrophysiotherapy treatment consisting of ultrasound, laser and interferential current techniques. Group 2 (*n* = 30) received USGET. Both groups did the same standardized eccentric exercise program. Periodic assessments of the subjects were carried out with the Victorian Institute of Sport Assessment-Patella (VISA-P) score. An analysis of means and a survival study were performed.

**Results:**

There were statistically significant differences in the VISA-P between the baseline and final follow-up in each treatment group. Group 1 (conventional electrophysiotherapy) went from 52.5 ± 18.8 to 61.9 ± 13.7 (in VISA-P < 90 subgroup) and from 69.1 ± 9.1 to 95.2 ± 2.5 (in VISA-P > 90 subgroup). Group 2 (USGET) went from 51.4 ± 17.9 to 63.3 ± 14.3 (in VISA-P < 90 subgroup) and from 66.3 ± 13.1 to 97.1 ± 1.7 (in VISA-P > 90 subgroup). There were statistically significant correlations between the baseline and final score in the VISA-P > 90 subjects upon completing the study but no statistically significant correlations between subjects with VISA-P < 90. The mean number of sessions applied was 22.6 ± 2.5 in Group 1 and 3.2 ± 0.9 in Group 2. The success probability in Group 1 was 36.1% versus 72.4% in Group 2. The difference was statistically significant.

**Conclusion:**

The results obtained with the combination of USGET and eccentric exercise reported better outcomes than with the conventional electrophysiotherapy techniques in the treatment of patellar tendinopathy.

## Background

Patellar tendinopathy has a high prevalence rate among athletes (Larsson et al., 2012). This is especially so in sports that involve repetitive movements that cause an overload of the tendon like jumping, changes of pace and direction as well as racing and pedalling. It appears in both professional athletes and amateurs (Childress and Beutler, 2013). Classically, the term tendinitis was used considering that the fundamental lesion was an inflammation of the tendon. Today, we know, histologically speaking, that we can find degenerated tissue with fragmentation, an alteration of the collagen and vascular hyperplasia, with inflammatory cells almost absent (Cook and Purdam, 2009; Khan et al., 2002).

This increased understanding of the pathophysiology leads to a change in the therapeutic approach. At present, the therapeutic techniques used in the treatment of tendinopathy have abandoned the goal of eliminating inflammation of the tendon and instead try to impact on the biology of the tendon to stimulate its regeneration (Cook and Purdam, 2009).

Different therapeutic options for tendinopathy can be found in the current literature. None of them has been clearly established as the gold standard and the best option is still a matter of debate (Andres and Murrell, 2008; Childress and Beutler, 2013). Since the concept of tendinopathy as a defective healing process has broadened, therapeutic options have been progressively moving toward restoring natural tendon biology. Within the therapeutic arsenal, eccentric exercises play an important role although they have been proven insufficient when the tissue is significantly degenerated (Visnes et al., 2005). These exercises assist in the recovery of the biomechanical qualities of the tendon if the biology of previously damaged tissue can be restored (Childress and Beutler, 2013).

The USGET is a minimally invasive treatment with reported good clinical results in the medium (Abat et al. 2014a, b) and long-term follow-up (Abat et al., 2015). This technique consists in an ultrasound-guided application of a galvanic electrolytic current that causes a controlled local inflammatory process in the target tissue. This allows for phagocytosis and the subsequent regeneration of the affected tissue (Abat et al. 2014a, b).

The aim of this study was to determine whether the application of USGET and eccentric exercise in patellar tendinopathy reported better results than those obtained with conventional physiotherapy treatment in terms of pain and function.

## Methods

### Design

The subjects were randomly assigned to each group by a computer-generated number table (deterministic algorithm). An external assistant generated the tables and assigned the patients to the appropriate treatments group. Based on this statistical stratification, they were divided into two groups: The electro-physiotherapy group (Group 1) and USGET group (Group 2). The evaluator did not know this at any times. The patients included in the study were identified by a numerical code after signing informed consent. Within each group, subjects were divided based on the Victorian Institute of Sport Assessment-Patella (Visa-P) final score as a Visa-P <90 or Visa-P ≥ 90. Systematic assessments were performed every two weeks during follow-up. Subjects received treatment for 2 months or until the symptoms were not present (VISA-P value ≥ 90).

The study was carried out in accordance with the international standards for clinical trials, the declaration of Helsinki and the Good Clinical Practice Regulations. The study protocol was reviewed and approved by the Reference Ethics Committee (n° 201000005507). All patients who fulfilled the inclusion criteria signed informed consent for study inclusion.

### Participants

Sixty-four patients were initially selected from the outpatient clinic to participate in the clinical trial. The inclusion criteria were being between 20 and 60 years of age, a clinical and ultrasound diagnosis of unilateral insertional patellar tendinopathy, having symptoms for more than one month and being athletically active before injury. The exclusion criteria were a prior knee surgery, associated lower limb injuries (like an anterior cruciate ligament injury or meniscopathy) or having received local steroid injections in the tendon prior to the study. Patients who took fluoroquinolones, anticoagulants or anti-inflammatory drugs were also excluded.

The study was composed of 32 subjects in Group 1 (24 males and 8 females with a mean age of 30.9 ± 5.9 years) and 32 subjects in Group 2 (27 men and 5 women with a mean age of 31.2 ± 6.5). There were four losses during follow up, two from each group, due to non-adherence to the treatment program. Thus, 60 subjects completed the study. Both groups were comparable with no statistical differences in any of the study variables (Table 1).

**Table 1:** Demographic and clinical data of participants separated by treatment group

	Group 1 (Electro-physiotherapy)	Group 2 (USGET)	*p.* value
Age (years)^a^	30.9 (5.9)	31.2 (6.5)	0.891
Sex (male:female)^b^	24:8	27:5	0.351
Weight (Kg)^a^	71.5 (11.2)	73.2 (11.1)	0.547
Height (m)^a^	174.7 (7.4)	175.8 (6.2)	0.501
BMI (kg/m2)^a^	23.3 (2.1)	23.6 (2.4)	0.631
Physical activity (days/week)^a^	3.8 (1)	4.3 (1.4)	0.055
Physical activity (hours/day)^a^	1.9 (1.5)	2.1 (1.2)	0.657
Laterality (Right:Left)^b^	24:8	23:9	0.719
Symptoms duration (months)^a^	29.5 (31.5)	28.8 (32.4)	0.929
# of previous episodes pain^a^	3.3 (2.3)	3.7 (2.6)	0.543
Time from the start of the last episode (months) ^a^	2.2 (0.9)	2.8 (2.9)	0.277
Thickening of the tendon (Yes: No) ^b^	32:0	32:0	1.000
Vascularization (Yes: No) ^b^	23:9	22:10	1.000

^a^ Statistics: Mean (standard deviation); *p* = Student t test. ^b^ Frequencies, *p* = Chi square

### Intervention

Eccentric exercises were performed in both groups in combination with either standard electrotherapy (Group 1) or USGET (Group 2). The subjects in Group 1 passed through an electrophysiotherapy sessions of 50 min for three days a week over 8 weeks. Each session saw Ultrasound (Endomed 982) on the patellar tendon that was pulsed (1:5) for 2 milliseconds at a frequency of 100 Hz and an intensity of 0.5 W/cm2 for 10 min. Laser CO_2_ (Asa Medical Laser) was also implemented with a fan shaped cannon over the surface of the patellar tendon with an energy of 15 joules at a potency of 10 watts for 2 min and Interferential Currents (Endomed 982) in a tetrapolar application at a frequency of 80–100 Hz for 15 min. Finally, eccentric exercises based on those described for the conservative treatment of patellar tendinopathy were performed. A slow single-leg squat on an incline of 25° was done in 3 sets of 15 repetitions with a 3-min rest between sets. The exercise was conducted without an external load for 15 min.

The subjects in Group 2 underwent a treatment protocol consisting of USGET and eccentric exercises. The eccentric exercises were performed in the same manner as in Group 1. A USGET session was conducted every two weeks. USGET was performed with the patient supine with the knee flexed to 20° after the area had been disinfected with isopropanol. The galvanic electrolytic current was applied with a sterile 0.25x25 millimetre stainless steel acupuncture needle. This procedure was performed with ultrasound guided puncturing in the superficial paratendon, deep paratendon and the intratendonous area at the inferior pole of the patella in its deepest portion. In each of these locations, 3 punctures were made (without removing the needle from the skin) with an intensity of 2 milliamps until the injured area was completely debrided.

### Outcome measures

A clinical history was completed on the first visit in which personal, physical, socio-demographic data, the medical history and symptoms were collected. A colour Doppler ultrasound was done to confirm the diagnosis of insertional patellar tendinopathy and the Victorian Institute of Sport Assessment-Patella (VISA-P) score (Hernandez-Sanchez et al., 2011) to assess symptoms, function and the ability to perform sport was completed (Hernandez-Sanchez et al., 2014). The VISA-P questionnaire was evaluated at the start point and at the end of the treatment at 2 months. The VISA-P consists of 8 items, 6 of which are analogue-visual scales from 0 to 10 where 10 represent the optimum. The first 6 questions cover the parameters of pain and function in different activities while the last 2 questions assess the parameters of function and the ability to perform sport. The maximum score is 100 points and corresponds to an asymptomatic and fully functional subject while the minimum score is 0 points. Visentini showed that it is a reliable tool for measuring the evolution of the PT and is validated by the scientific community (Visentini et al. 1998). During the study, patients with VISA-P values of less than 90 points were considered “not healed or symptomatic” and over 90 points as “healed or asymptomatic”.

An orthopaedic surgeon skilled in ultrasound diagnosis performed the ultrasonography evaluation. A protocol defined by the European Society of Musculoskeletal Radiology from the Musculoskeletal Ultrasound Technical Guidelines for the Knee was used (Beggs et al., 2012). This bilateral comparative evaluation was performed with the patient supine, the knee positioned at 0° and 20° of flexion and with a longitudinal and transversal view of the patellar tendon from its proximal insertion on the patella to its distal insertion in the tibial tuberosity. The parameters studied for diagnosis of patellar tendinopathy were: thickening of the tendon, the presence of an intra-tendinous hypoechoic areas, the presence or absence of irregularities in the cortical bone of the distal part of the patella and the presence or absence of intra-tendinous calcifications or hipervascularization.

### Data analysis

The results are expressed as means, standard deviations (SD) and a confidence intervals of 95%. A *p*-value of less than 0.05 was considered statistically significant. A Kolmogorov normality test was done for the comparison study between the evaluation variables.

In cases of non-normality and asymmetries in the distributions of data variables, nonparametric tests (Mann–Whitney or Wilcoxon) were used. The comparison and correlation study of VISA-P scores between the final and baseline in each treatment groups and between asymptomatic and symptomatic subjects in the follow-up study was performed with the Student's T-tests and Pearson correlations. A study of survival with the Kaplan-Meier method, comparing the survival curves in each of the treatment groups with the Mantel-Haenszel test (log-rank), was done. The probability of success of each treatment was calculated and compared. The statistical power was 99.9%. Statistical analysis was performed using the SPSS 15 package (SPSS Inc., Chicago, Illinois).

## Results

### Research questions

**Fig. 1:**
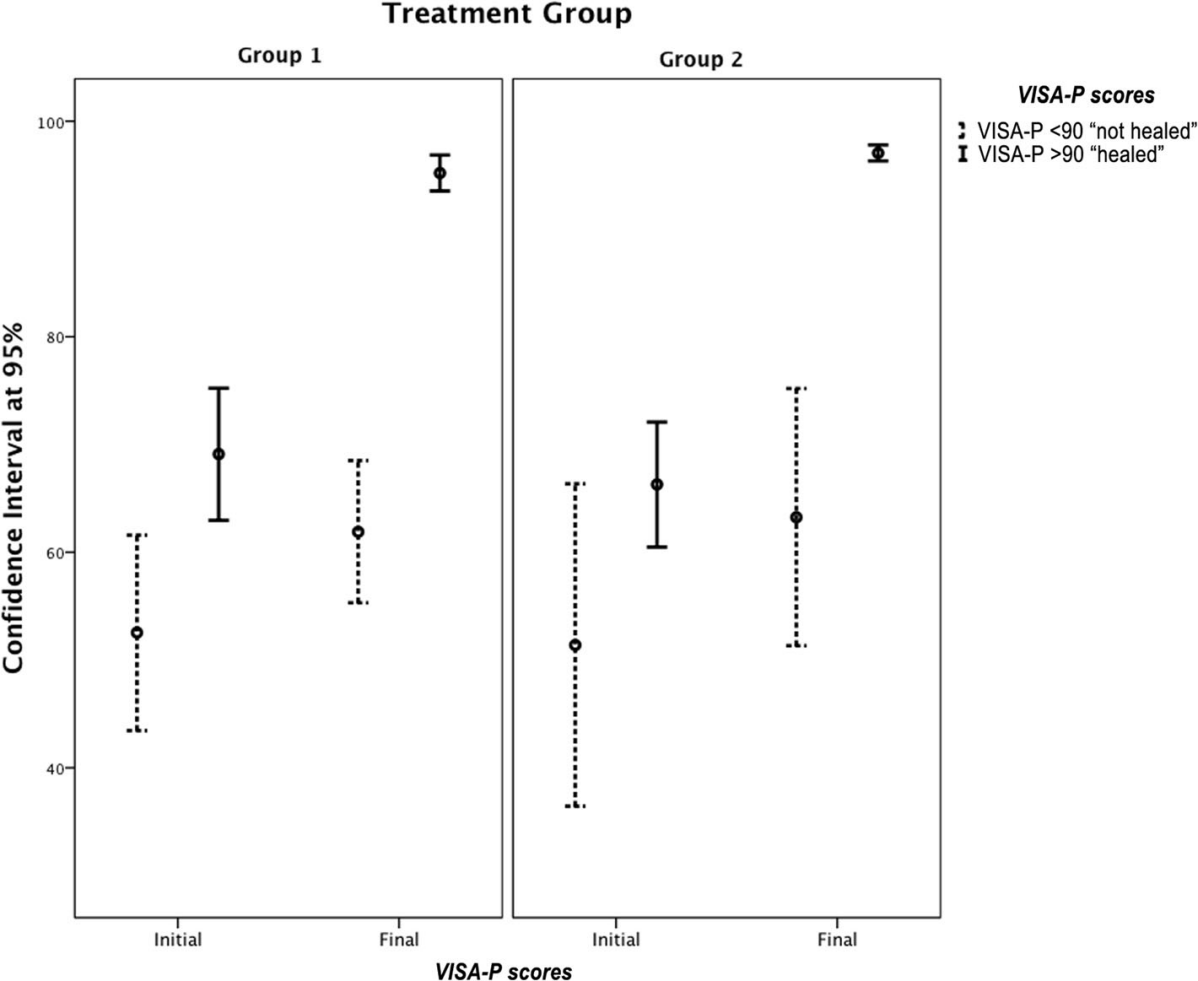
Error bar chart for the confidence interval at 95% at the start and end VISA-P scores in each treatment group. Subjects ranked as VISA-P > 90 and VISA-P < 90

The functional assessment according to the VISA-P showed statistically significant differences (*p* <0.05) between the initial and final assessment (Table 2) in the subjects with a VISA-P <90 (a difference of 10.1 points [95%CI 6.3 to 13.8]) and the ones with a VISA-P ≥ 90 (a difference of 29.2 points [95%CI 13.37 to 24.7]). These differences remained significant when analysing the results by groups (Fig. 1).

**Table 2:** Score on the VISA-P scale at the initial and final evaluation by treatment Group

		VISA-p Initial Eval.^a^	VISA-p Final Eval.^a^	*p*. value^b^
Group 1 (Electro-physiotherapy) *n* = 30	VISA-p < 90 *n* = 19	52.5(18.8) [43.5-61.6]	61.9 (13,7) [55.3–68.5]	*p* < 0.001
	VISA-p > 90 *n* = 11	69.1 (9.1) [62.9-75.2]	95.2 (2.5) [93.5-96.9]	*p* < 0.003
Group 2 (USGET) *n* = 30	VISA-p < 90 *n* = 8	51.4 (17.9) [36.4-66.3]	63.3 (14.3) [51.3-75.2]	*p* < 0.021
	VISA-p > 90 *n* = 22	66.3 (13.1) [60.5-72.1]	97.1 (1.7) [96.3-97.8]	*p* < 0.001
TOTAL *n* = 60	VISA-p < 90 *n* = 27	52.2 (18.2) [44.9-59.4]	62.3 (13.6) [56.9-67.7]	*p* < 0.001
	VISA-p > 90 *n* = 33	67.2 (11.2) [63.0-71.4]	96.4 (2.1) [95.7-97.2]	*p* < 0.001

^a^ Victorian Institute of Sport Assessment-Patella (VISA-P) values expressed as mean (±SD) and [coefficient interval]. ^b^ *p* = non-parametric Wilcoxon test

Ranked as not healed (VISA-P < 90) and healed (VISA-P ≥ 90) at the final follow-up

**Fig. 2:**
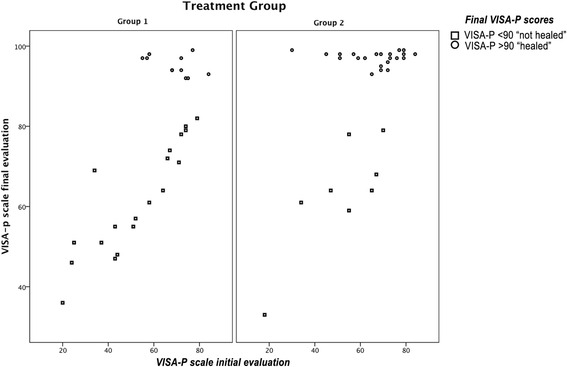
Scatter plot between the scores at the start and end VISA-P in each treatment group. It distinguishes subjects with VISA-P > 90 and VISA-P < 90

A correlation analysis was performed to study the relationship between the VISA-P scores at baseline and those at the last evaluation (the 5th evaluation or in those who were considered "cured") in all the subjects. For the total sample, a positive association between the initial and final VISA-P (*n* = 60; r2 = 0,457; *p* = 0,000) was observed. However, we observed different behaviors upon making correlations based on whether they were considered "healed or asymptomatic" or "not healed or symptomatic" at the last evaluation. In subjects with a final VISA-P ≥ 90, there were no statistically significant differences for either the total sample (*n* = 33) or for each of the intervention groups, 11 patients for electro-physiotherapy and 22 for USGET. For subjects with a VISA-P <90 at the end of the study, there were statistically significant differences (Fig. 2 and Table 3).

**Table 3:** Correlation analysis for the whole series

CORRELATIONS				
				VISA-P Last Eval.
TOTAL	Group 1 (Electro-physiotherapy)	VISA-P Initial Eval.	(r)	0,774
			(r^2^)	0,599 (59,9%)
			*p* (value)	0,000
	Group 2 (USGET)	VISA-P Initial Eval.	(r)	0,57
			(r^2^)	0,325 (32,5%)
			*p* (value)	0,001
	TOTAL	VISA-P Initial Eval.	(r)	0,676
			(r^2^)	0,457 (45,7%)
			*p* (value)	0,000
VISA-P < 90 Final Eval.	Group 1 (Electro-physiotherapy)	VISA-P Initial Eval.	(r)	0,88
			(r^2^)	0,774 (77,4%)
			*p* (value)	0,000
	Group 2 (USGET)	VISA-P Initial Eval.	(r)	0,818
			(r^2^)	0,669 (66,9%)
			*p* (value)	0,013
	TOTAL	VISA-P Initial Eval.	(r)	0,859
			(r^2^)	0,738 (73,8%)
			*p* (value)	0,000
VISA-P ≥ 90 Final Eval.	Group 1 (Electro-physiotherapy)	VISA-P Initial Eval.	(r)	−0,491
			(r^2^)	0,241 (24,1%)
			*p* (value)	0,125
	Group 2 (USGET)	VISA-P Initial Eval.	(r)	−0,136
			(r^2^)	0,018 (1,8%)
			*p* (value)	0,548
	TOTAL	VISA-P Initial Eval.	(r)	−0,262
			(r^2^)	0,069 (6,9%)
			*p* (value)	0,140

Victorian Institute of Sport Assessment-Patella (VISA-P). Pearson Correlation Coefficient (r). Coefficient of Determination (r2) (% of Variance Explained). *p* = non-parametric Wilcoxon test

The number of sessions is not comparable between treatment groups due to the fact that the frequency in each case was different. In Group 1, an average 22.6 ± 2.5 sessions were performed, while 3.2 ± 0.9 USGET application sessions were needed in Group 2. There were no statistically significant differences in terms of the time in treatment between groups (Table 4). No adverse events were found in either group during the study.

**Table 4:** Number of sessions and duration of treatment by Group and ranked as not healed (VISA-p < 90) or healed (VISA-p ≥ 90) at the final follow-up

	GROUP 1 (Electro-physiotherapy) *n* = 30			Group 2 (USGET) *n* = 30		
	Not healed *n* = 19	Healed *n* = 11	TOTAL *n* = 30	Not healed *n* = 8	Healed *n* = 22	TOTAL *n* = 30
NUMBER SESSIONS^a^	24(0) [24–24]	36.3(5.04) [32.9–39.7]	38.7(3.5) [37.3–39.9]	4(0) [4–4]	3.0(0.9) [2.6–3.4]	3.3(0.9) [2.9–3.6]
TREATMENT TIME (Days)^a^	56(0) [56–56]	50.9(7.1) [46.1–55.6]	54.1(4.8) [52.3–55.9]	56(0) [56–56]	42.0(13.6) [35.9–48.0]	45.7(13.2) [40.8–50.6]

^a^ Statistics: Mean (standard deviation) [95% confidence interval]. No statistically significant differences were seen when comparing the number of sessions or time of treatment used in the comparison of both groups

**Fig. 3:**
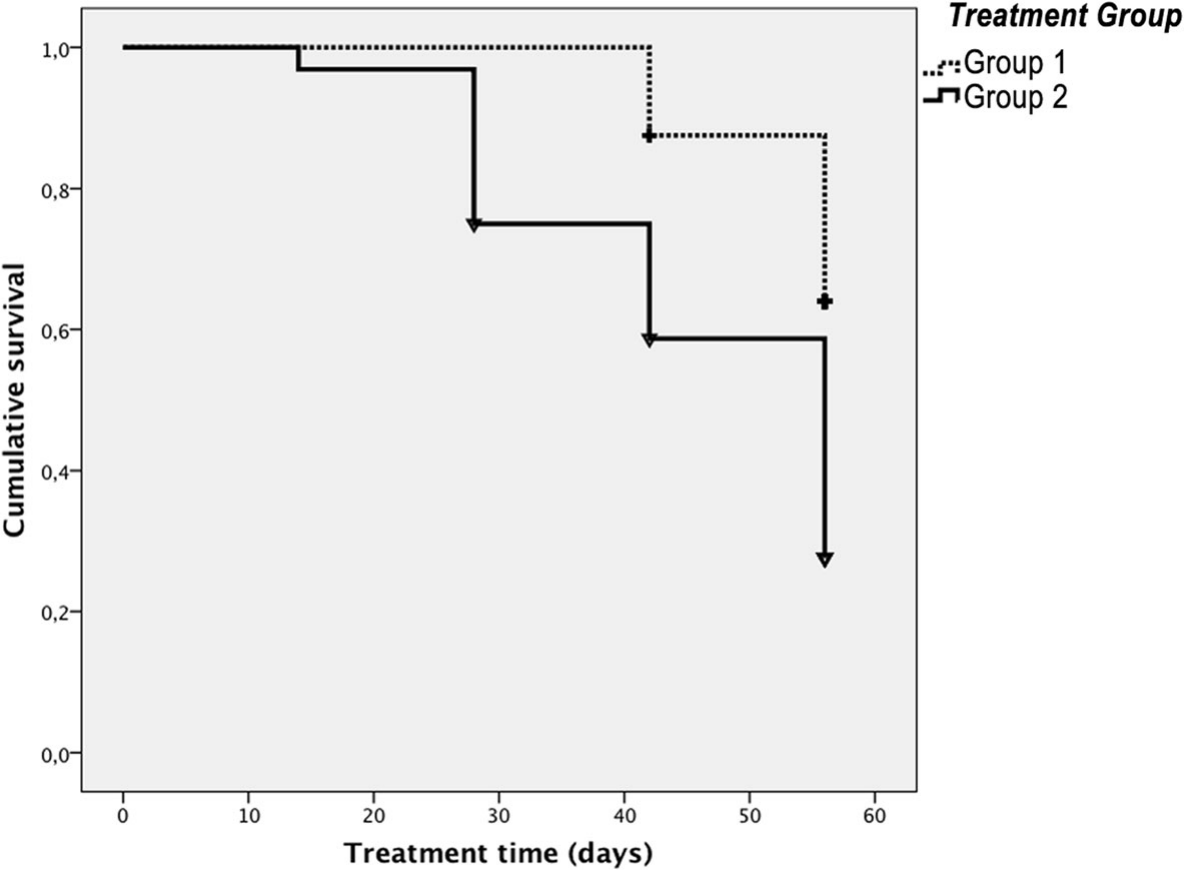
Cumulative survival graph in each of the treatment groups, VISA-P > 90 being the event studied

The survival analysis showed that patients who received conventional electro-physiotherapy had a 36.1% chance of success versus 72.4% of the group treated with USGET at the end of the follow up period. In the survival analysis, the fact that subjects had a VISA-P ≥ 90 ("cured") was considered an event of interest (cutoff). At that point, regardless of the evaluation, the follow up period in the study terminated. That is, Group 2 showed a 36.3% greater heal rate [95% CI 36.1 to 36.5] at the final follow-up than Group 1. This difference was statistically significant (χ^2^ = 10.312; df = 1; *p* = 0.001). In Group 2, 50% of subjects healed at between 28 and 56 days, somewhere between the second and the fourth USGET sessions. At 42 days, the probability of treatment success in Group 1 was 12.5% compared to 58.7% for Group 2 (Fig. 3).

## Discussion

The results of this study using the VISA-P score evaluation show that the use of USGET and eccentric exercise is more effective in dealing with patellar tendinopathy than treatment with conventional electro-physiotherapy.

One explanation for the difference in efficacy between the treatments might be in the pathophysiological process of tendinopathy. Chronic pathologies are histologically characterized by tendon tissue degeneration with failure in the repair response in which hyperplasia and pathological neovascularization fibroblasts have been seen (Cook and Purdam, 2009; Khan et al., 2002). Alfredson et al. (Alfredson et al., 2003) suggested that these new vessels and nerves that accompany them were involved in the mechanisms of tendinopathy pain but the answer to the origin of the pain is an issue that is still undetermined.

The lower prevalence of healing in Group 1 suggests that addressing patellar tendinopathy with the conventional electro-physiotherapy techniques studied should not be the basis of the strategy for the treatment for this disease. This approach is justified in the literature. Although there are some studies that have been published on the benefits of pain and soft tissue regeneration by applying laser (Bjordal et al., 2006), ultrasound (Fu et al., 2008) or electro-therapy (Chang et al., 2015), many more authors have concluded that there is no scientific evidence to attribute any clinical significance to these techniques in the treatment of tendinopathy (Andres and Murrell, 2008; Leadbetter, 2005).

The results show that the USGET could be a technique capable of acting on tendon biology by destroying the degenerated tissue and causing an inflammatory response that could trigger the biological process of collagen repair (Abat et al. 2014a, b). The only technique in common in the two intervention groups was eccentric exercise. The benefits of eccentric training in tendinopathy have been extensively studied (Jonsson and Alfredson, 2005; Young et al., 2005; Larsson et al., 2012; Visnes and Bahr, 2007). An effect on the biomechanics of the tendons is attributed to it, producing a stimulus in the voltage load and stretching, which are required in directing the orientation of collagen in the process of proliferation and maturation. However, with the application of the same exercise protocol in both groups, the cure rate was different. Therefore, the better results in Group 2 cannot be attributed to eccentric training only as one would expect a similar outcome in Group 1. The explanation for this greater effectiveness might be based on the combined application of a technique capable of eliciting a regenerative response a priori in tendinopathy as is USGET. It would be followed by another that would cause sufficient mechanical stimulation of the tendon tissue, producing a positive effect on cellular activity and the restructuring of the extracellular matrix.

The work has some limitations, including the fact that the study has divided the final score obtained on the scale VISA-P into two categories; VISA-P < 90 and VISA-P ≥ 90. Patients within the first category (VISA-P < 90) were considered as having symptoms and functional deficits and those in the second (VISA-P ≥ 90) were considered asymptomatic. Based on the literature, it is difficult to establish a clear boundary for the normal value in the VISA-P score and it is also very difficult to establish the score that a subject must have to consider them completely asymptomatic (Visnes and Bahr, 2007; Jonsson and Alfredson, 2005). Furthermore, failure to present symptoms or have no functional deficits implies that the structure and morphology of the tendon has been completely restored to normal (Coleman et al., 2000). We still consider the division into these categories to be helpful in understanding the results.

On the other hand, an ultrasound assessment of changes that the applied treatment might produce is not included at the end of the study. However, a morphological picture is not predictive of the symptoms of the patellar tendon (Warden et al., 2007) and ultrasound imaging is unable to distinguish changes caused by short-term treatment (Coleman et al., 2000).

An important limitation might be that various treatments were carried out together. Nevertheless, that fact represents the reality in current treatment protocols for tendinopathies.

Another limitation of the study is the short symptom duration before the treatment (1 month) and the relative short follow-up time. Moreover, other randomized trials have used intervention periods of equal length or even shorter (Stasinopoulos and Stasinopoulos, 2004). Additionally a longer duration of symptoms before treatment may have had an influence on the results. However, previous studies of this scientific group have shown that the effectiveness of the proposed treatment protocol is maintained even during much longer periods of symptomatology (Abat et al. 2014a, b). Despite all this, it is possible that a period of longer intervention might affect the results. Therefore, future studies will be necessary.

## Conclusion

The results obtained with the combination of USGET and eccentric exercise have reported better outcomes than conventional electro-physiotherapy techniques in the treatment of patellar tendinopathy.

## Abbreviations

SD: Standard deviations
USGET: Ultrasound-guided galvanic electrolysis technique
Visa-P: Victorian Institute of Sport Assessment-Patella.
